# Type I Interferonopathy among Non-Elderly Female Patients with Post-Acute Sequelae of COVID-19

**DOI:** 10.3390/v16091369

**Published:** 2024-08-28

**Authors:** Donghua Xu, Xuebin Qin

**Affiliations:** 1 Division of Comparative Pathology, Tulane National Primate Research Center, Tulane University School of Medicine, Tulane University, 18703 Three Rivers Road, Covington, LA 70433, USA; flower322@163.com; 2 Department of Microbiology and Immunology, School of Medicine, Tulane University, New Orleans, LA 70112, USA

**Keywords:** post-acute sequelae of COVID-19, type I interferon signaling, non-elderly, females, signal transducers and activators of transcription 2

## Abstract

The pathophysiological mechanisms of the post-acute sequelae of COVID-19 (PASC) remain unclear. Sex differences not only exist in the disease severity of acute SARS-CoV-2 infection but also in the risk of suffering from PASC. Women have a higher risk of suffering from PASC and a longer time to resolution than men. To explore the possible immune mechanisms of PASC among non-elderly females, we mined single-cell transcriptome data from peripheral blood samples of non-elderly female patients with PASC and acute SARS-CoV-2 infection, together with age- and gender-matched non-PASC and healthy controls available from the Gene Expression Omnibus database. By comparing the differences, we found that a CD14^+^ monocyte subset characterized by higher expression of signal transducers and activators of transcription 2 (STAT2) (CD14^+^STAT2^high^) was notably increased in the PASC patients compared with the non-PASC individuals. The transcriptional factor (TF) activity analysis revealed that STAT2 and IRF9 were the key TFs determining the function of CD14^+^STAT2^high^ monocytes. STAT2 and IRF9 are TFs exclusively involving type I and III interferon (IFN) signaling pathways, resulting in uncontrolled IFN-I signaling activation and type I interferonopathy. Furthermore, increased expression of common interferon-stimulated genes (ISGs) has also been identified in most monocyte subsets among the non-elderly female PASC patients, including IFI6, IFITM3, IFI44L, IFI44, EPSTI1, ISG15, and MX1. This study reveals a featured CD14^+^STAT2^high^ monocyte associated with uncontrolled IFN-I signaling activation, which is indicative of a possible type I interferonopathy in the non-elderly female patients with PASC.

## 1. Introduction

Coronavirus disease 2019 (COVID-19) also causes long-term symptoms persisting for several months or even years, termed post-acute sequelae of COVID-19 (PASC) or long COVID [1,2]. During the past 4 years of the global COVID-19 pandemic, PASC has affected more than millions of people [3]. PASC can manifest in people across the life span. The pathophysiological mechanism of PASC remains unclear. Moreover, PASC probably has several subtypes with different risk factors and pathophysiological mechanisms [1]. There is a sex discrepancy regarding the disease severity of acute SARS-CoV-2 infection, with men suffering from a higher disease burden and more intensive care unit (ICU) admissions than women. On the contrary, women have a higher risk of suffering from PASC than men [4,5,6,7]. Sex differences in PASC are featured, with women suffering from more PASC and a longer time to resolution. Apart from gender, age-specific differences exist in the disease severity of acute SARS-CoV-2 infection, with the elderly suffering from a higher disease burden. Unlike acute SARS-CoV-2 infection, middle-aged individuals (30–65 years) are more likely to suffer from PASC than older ones [4,7]. Therefore, middle-aged women have an increased risk of PASC. Nonetheless, the causes and potential mechanisms underlying PASC among non-elderly female patients are not fully understood.

To explore the potential immune mechanisms underlying the pathophysiological processes of PASC among non-elderly females, we mined all available single-cell RNA-sequencing (scRNA-seq) data from the GEO database (https://www.ncbi.nlm.nih.gov/geo/) (accessed on 20 February 2024). We compared the single-cell transcriptome data of peripheral blood samples of non-elderly female patients with PASC and acute SARS-CoV-2 infection, together with age- and gender-matched non-PASC and healthy controls available in the database. We identified a featured CD14^+^STAT2^high^ monocyte associated with uncontrolled IFN-I signaling activation, which is indicative of a possible type I interferonopathy in the non-elderly female patients with PASC. Our results provide novel insight into the development of new therapies for PASC.

## 2. Methods

### 2.1. Single-Cell Transcriptome Data

We retrieved the scRNA-seq data from the Gene Expression Omnibus (GEO) database (https://www.ncbi.nlm.nih.gov/geo/). We included all eligible scRNA-seq data of whole blood or peripheral blood mononuclear cells (PBMC) from patients with long COVID or acute SARS-CoV-2 infection and controls from non-PASC or healthy individuals for our analyses. The inclusion criteria were as follows: long COVID patients were diagnosed at least 5 months after onset and continued to experience persistent symptoms such as fatigue, dyspnea, or cognitive impairment. For the healthy control group, we selected a similar number of healthy controls matched with age and gender from individuals without a history of acute COVID-19. The scRNA-seq datasets that did not meet the above-mentioned inclusion criteria were excluded.

### 2.2. Data Download and Alignment

We obtained the raw sequencing data of the selected datasets in SRA format through downloading links provided by the GEO database. SRA files were converted into FASTQ files via fasterq-dump. Cell Ranger 7.2.0 software from 10× Genomics was used for quality control, alignment, and transcript quantification of the FASTQ data. During alignment, FASTQ files were aligned to GRCh38-2020, and introns model was used. We assessed the quality of each sample, including metrics such as median genes per cell, fraction reads in cells, and number of cells.

### 2.3. The scRNA-seq Transcriptome Analysis

Downstream analyses of scRNA-seq transcriptome data were performed using Seurat V5.0.2. The gene expression matrixes of all samples were integrated with a combination of harmony and canonical correlation analysis (CCA). Cell clustering, dimensionality reduction, and visualization were then performed, and the number of principal components (PCs) was 30. Cell types were identified based on the highly expressed genes in each cluster. The visualization of immune cell clusters was based on the dimension reduction method of uniform manifold approximation and projection (UMAP). Candidate feature genes for specific subsets were identified with the find all markers function in Seurat. Differential gene expression analysis was conducted to estimate the differentially expressed genes (DEGs) between long COVID patients, non-COVID controls, acute COVID-19 patients, and healthy controls. A cut-off of log2 fold change (log2FC) of ≥0.2 or ≤−0.2 and a Benjamini adjusted P value less than 0.01 were applied to identify DEGs.

### 2.4. Functional Enrichment Analysis

Functional enrichment analysis was performed based on genes with high expression in cell subtypes or genes with inter-group differential expression to explore significantly enriched biological processes and pathways altered in long COVID patients or cell subsets. Functional annotation of the signature genes was performed with cluster profiler. Gene Ontology-Biological Process (GO-BP) gene sets were mainly analyzed in the functional enrichment analyses. Gene sets with a false discovery rate (FDR) q value less than 0.05 were considered to have significantly enriched pathways.

### 2.5. Transcription Factor (TF) Analysis

DoRothEA can infer the functional status and activities of TF in cells from the gene expression data of single cell. We used DoRothEA to analyze the key TFs to determine the functional status of cell subset. The changes in TF activities in cells between different groups were also analyzed.

### 2.6. Statistical Analysis

Data were presented as mean or median with 95% confidence interval (CI). The inter-group differences were analyzed using t-test or chi-square test. GraphPad Prism (version 8.0) and R software (version 4.2.3) were used for data analysis. Differences were considered statistically significant with *p* < 0.05.

## 3. Results

### 3.1. Characteristics of Participants

This study included a total of 41 female participants, consisting of 15 acute COVID-19 infection patients, 15 healthy controls, eight PASC patients, and three non-PASC controls from seven publications [8,9,10,11,12,13,14]. The mean ages of acute COVID-19 infection, healthy controls, PASC, and non-PASC controls were 45.9, 48.7, 43.5, and 41.7 years old, respectively. The characteristics of the 41 female participants included in this study are shown in Table 1. The raw scRNA-seq data of PBMCs from all participants were all available in the NCBI SRA database.

### 3.2. Changes in Peripheral Immune Cells among Non-Elderly Female PASC

**Figure 1:**
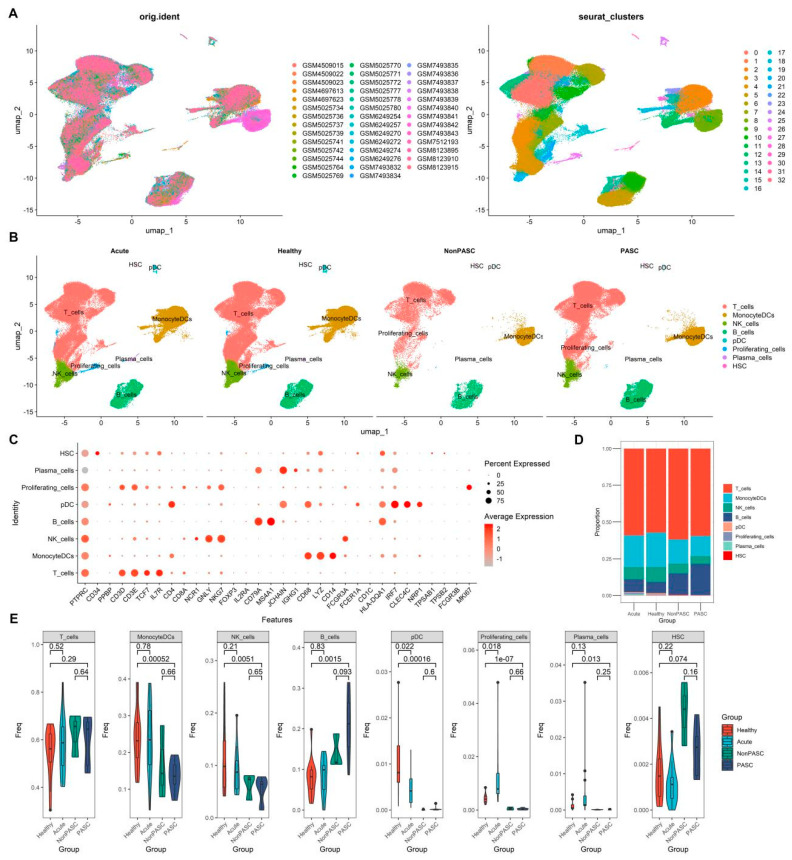
Single-cell transcriptome analyses of PBMCs from non-elderly female participants consisting of acute COVID-19 infection patients, healthy controls, PASC patients, and non-PASC controls ((**A**), Cluster distribution via UMAP of PBMCs of all participants, including those four groups that were grouped by sample (**Left**) or cell cluster (**Right**). (**B**), Cluster distribution via UMAP of PBMCs across four groups. (**C**), Dot plot shows the expression percentages and the expression levels of common markers of immune cells in different cell clusters in the analysis of PBMCs of all participants, including those four groups. (**D**), Comparison of the composition of major immune cells across four groups. (**E**), Difference in the proportions of major immune cells across four groups).

After integrating all sample datasets and quality control, a total of 207,563 immune cells were obtained from 41 scRNA-seq data of PBMC samples. Through clustering and gene expression patterns of cell markers, 32 distinct cell clusters were identified (Figure 1A). These clusters were further annotated for cell type using known cell markers. Most cell clusters were generally annotated as monocytes, B cells, T cells, and NK cells (Figure 1B,C). There was no obvious difference in the percentages of monocytes, B cells, T cells, and NK cells between PASC and non-PASC controls (Figure 1D,E). Furthermore, no significant difference was observed for the percentages of monocytes, B cells, T cells, and NK cells between acute COVID-19 infection and healthy controls (Figure 1D,E). However, obvious differences in the percentages of monocytes, B cells, and NK cells between PASC and healthy controls were found (Figure 1D,E), which were likely to be caused by bias from the non-biological batch effect or technical variation among different experiments. There was alsoa significant difference in the percentages of NK cells between non-PASC controls and healthy controls (Supplementary Figure S1), which were likely to be biased by non-biological batch effects. There were significant differences in the percentages of B cells, monocytes, and NK cells between PASC patients and acute COVID-19 infection cases, which may also be attributed to non-biological batch effects or technical variations across different study cohorts (Supplementary Figure S1). Therefore, we mainly used outcomes of the comparison between the PASC group and the non-PASC group, which were from the same study cohort and could exclude the impact of those non-biological batch effects, and outcomes of the comparison between the acute infection group and the healthy control group, which had a low risk of non-biological batch effects.

### 3.3. Changes in Peripheral Blood Monocytes among Non-Elderly Female PASC

**Figure 2:**
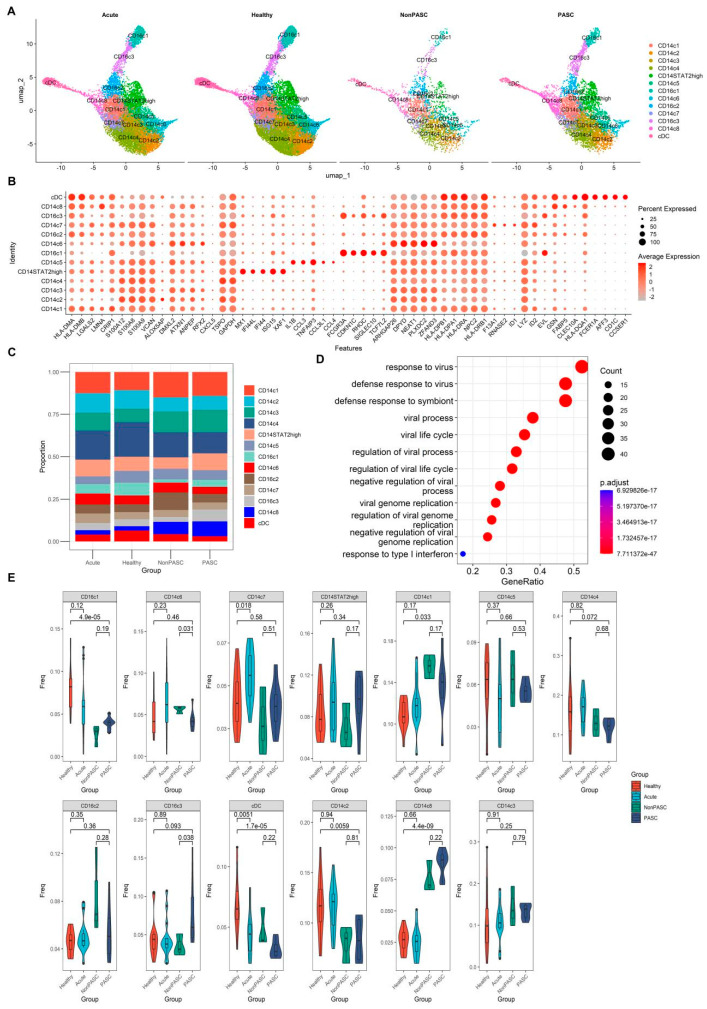
Single-cell transcriptome analyses of monocytes and cDCs from non-elderly female participants consisting of acute COVID-19 infection patients, healthy controls, PASC patients, and non-PASC controls ((**A**), Cluster distribution via UMAP of monocytes and cDCs across four groups. (**B**), Dot plot shows the expression percentages and the expression levels of key markers in different cell clusters in the analysis of monocytes and cDCs of all participants, including those four groups. (**C**), Comparison of the composition of cell clusters across four groups. (**D**), Enrichment plots of GO analysis of signature genes highly expressed in the cluster of CD14^+^STAT2^high^. (**E**), Difference in the proportions of cell clusters across four groups).

**Figure 3:**
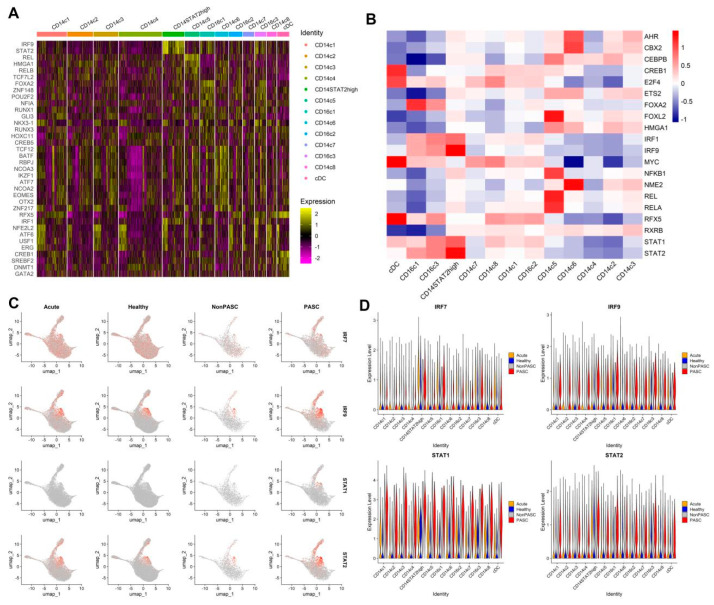
Comparison of TF activities in different cell clusters of monocytes and different groups ((**A**), Heatmap shows the activity scores of feature TFs in each cell of different cell clusters of monocytes. (**B**), Heatmap shows the mean activity scores of feature TFs in different cell clusters of monocytes. (**C**), Feature plots show the mean activity scores of key TFs among four different groups. (**D**), Violin plots show the expressions of key TFs in those cell subsets across four groups).

The peripheral blood monocytes and cDCs from those non-elderly females were further integrated with the CCA method. Through clustering and specific gene expression patterns of cell markers, 13 distinct cell clusters were identified (Figure 2A,B). There were nine CD14 monocyte subsets, three CD16 monocyte subsets, and one cDC subset. The expression of STAT2 in most monocyte clusters was obviously enhanced among PASC patients compared with non-PASC individuals (Figure 3). A CD14 monocyte subset, named the CD14^+^STAT2^high^ cluster, was increased modestly in the PASC patients when compared with non-PASC controls (Figure 2C,E). However, there was no statistical significance for the difference in the CD14^+^STAT2^high^ subset between PASC patients and non-PASC controls due to the insufficient sample size in this analysis (Figure 2E). A CD16 monocyte subset, named the CD16c3 cluster, was also increased in PASC patients when compared with non-PASC controls (Figure 2C,E). CD14c6 cluster was decreased in PASC patients when compared with non-PASC controls (Figure 2C,E). Functional enrichment analysis of the signature genes highly expressed in the cluster of CD14^+^STAT2^high^ revealed that pathways related to virus infection and type I interferon were significantly enriched (Figure 2D), suggesting a key role of the CD14^+^STAT2^high^ cluster in type I interferon responses. There were obvious differences in the percentages of some monocyte subsets between PASC and healthy controls, which was likely to be caused by the non-biological batch effect or technical variation among different experiments. The outcomes of the comparison of monocyte subset percentages between acute COVID-19 infection and PASC were shown in Supplementary Figure S1B, in which the differences may also be attributed to non-biological batch effects or technical variations across different study cohorts.

Analyses of TF activity in different cell clusters of monocytes revealed that STAT2 and IRF9 were the key TFs determining the function of CD14^+^STAT2^high^ monocytes, and aryl hydrocarbon receptor (AhR) and CBX2 were the key TFs determining the function of CD14c6 (Figure 3A,B). The activity of STAT2 and IRF9 in the CD16c3 cluster was also higher compared with other monocyte subsets, suggesting a key role of those two TFs in regulating the function of the CD16c3 cluster. The activity of STAT2 and IRF9 in both the CD14^+^STAT2^high^ cluster and some other monocytes increased in PASC patients when compared with non-PASC controls (Figure 3C). The expression of STAT2 in most monocyte clusters increased in PASC patients when compared with non-PASC controls (Figure 3D).

### 3.4. Increased Expression of Interferon-Stimulated Genes (ISGs) in Monocytes

**Figure 4:**
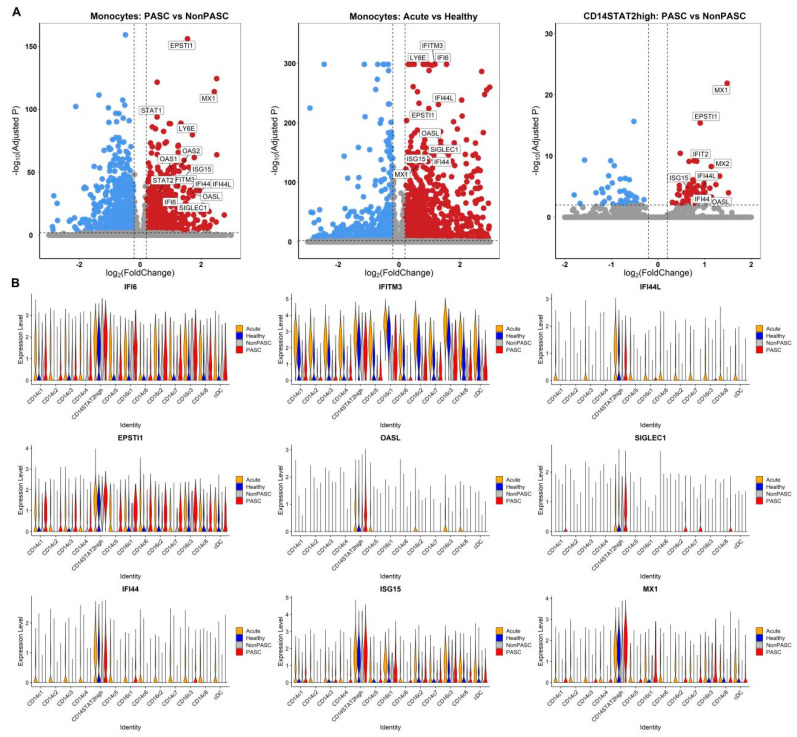
Increased expression of ISGs in most monocyte subsets among non-elderly female PASC patients ((**A**), Volcano plots for the DEGs in the monocytes between PASC patients and non-PASC controls, the DEGs in the monocytes between acute COVID-19 infection patients and healthy controls, and the DEGs in the CD14^+^STAT2^high^ cluster between PASC patients and non-PASC controls, respectively. The x axes represent the log2 of fold change in the gene expression between the comparison groups, and the y axes represent the −log10 of Benjamini–Hochberg adjusted *p* values. Genes with a Benjamini–Hochberg adjusted *p* < 0.01 and an absolute value of log2(fold change) > 0.2 were deemed to be DEGs. Up-regulated DEGs are shown in red, while down-regulated DEGs are shown in blue. (**B**), Violin plots show the expressions of common interferon-stimulated genes such as IFI6, IFI44L, MX1, ISG15, and IFIT3 in those monocyte subsets across four groups).

We compared the single-cell transcriptome differential expression between PASC patients and non-PASC controls (Figure 4). Many genes were significantly differentially expressed between PASC patients and non-PASC controls, including some common ISGs, including IFI6, IFITM3, IFI44L, IFI44, EPSTI1, ISG15, and MX1 (Figure 4A). Increased expressions of common ISGs in most monocyte subsets among non-elderly female PASC patients were identified (Figure 4B). Therefore, the type I interferon pathway was activated in PASC among non-elderly females, which was highly related to the pathophysiological processes of PASC.

### 3.5. The scRNA-Seq Transcriptome Analysis of B Cells

**Figure 5:**
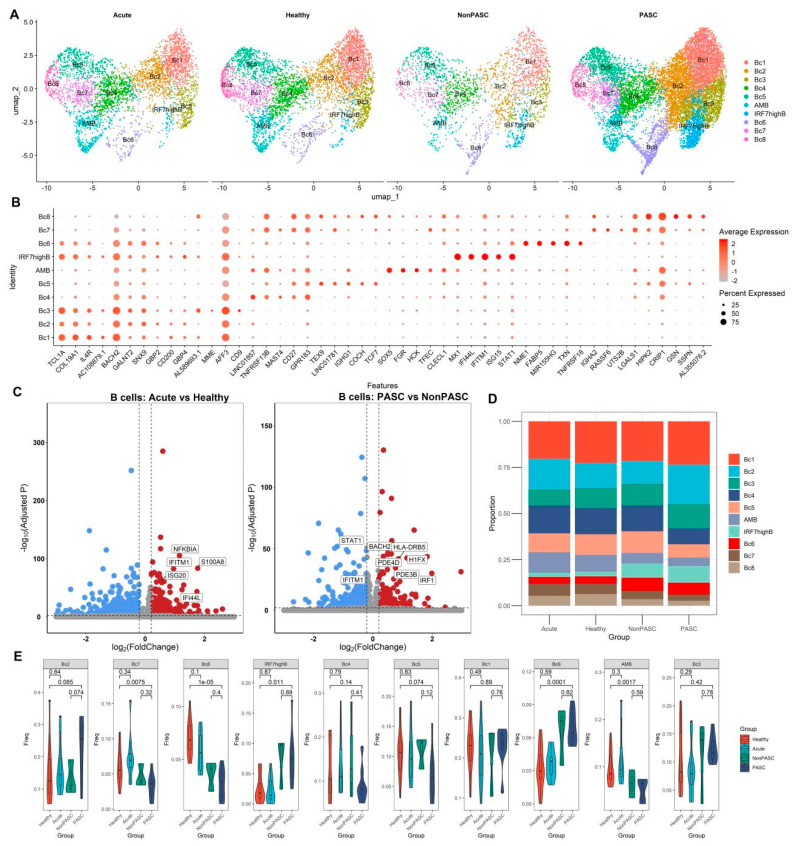
Single-cell transcriptome analyses of B cells from non-elderly female participants consisting of acute COVID-19 infection patients, healthy controls, PASC patients, and non-PASC controls ((**A**), Cluster distribution via UMAP of B cells across four groups. (**B**), Dot plot shows the expression percentages and the expression levels of key markers in different cell clusters. (**C**), Volcano plots for the DEGs in the B cells between acute COVID-19 infection patients and healthy controls, and the DEGs in the B cells between PASC patients and non-PASC controls, respectively. Genes with a Benjamini–Hochberg adjusted *p* < 0.01 and an absolute value of log2(fold change) > 0.2 were deemed to be DEGs. Up-regulated DEGs are shown in red, while down-regulated DEGs are shown in blue. (**D**), Comparison of the composition of cell clusters across four groups. (**E**), Difference in the proportions of cell clusters across four groups).

B cells from those non-elderly female participants were further integrated with the CCA method. Through clustering and gene expression patterns of cell markers, nine distinct cell clusters were identified (Figure 5A,B). We compared single-cell differential expression between PASC patients and non-PASC controls (Figure 5C). Many genes were significantly differentially expressed in B cells between PASC patients and non-PASC controls, but few ISGs were differentially expressed in B cells between PASC patients and non-PASC controls (Figure 5C). There was no significant difference in the percentages of those B cell subsets between PASC and non-PASC controls (Figure 5D,E). Although there was an obvious difference in the percentages of those B cell subsets between PASC and healthy controls (Figure 5D,E), it was likely to be caused by the non-biological batch effect. The outcomes of the comparison of B cell subset percentages between acute COVID-19 infection and PASC were shown in Supplementary Figure S2A, in which the differences may also be attributed to non-biological batch effects or technical variations across different study cohorts.

### 3.6. The scRNA-Seq Transcriptome Analysis of T Cells

**Figure 6:**
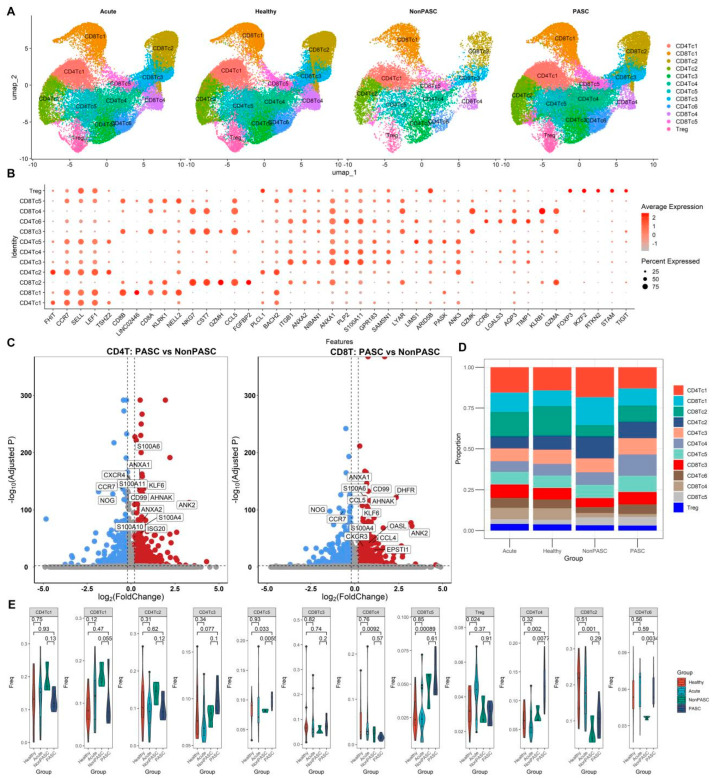
Single-cell transcriptome analyses of T cells from non-elderly female participants consisting of acute COVID-19 infection patients, healthy controls, PASC patients, and non-PASC controls ((**A**), Cluster distribution via UMAP of T cells across four groups. (**B**), Dot plot shows the expression percentages and the expression levels of key markers in different cell clusters. (**C**), Volcano plots for the DEGs in the CD4^+^ T cells and CD8^+^ T cells between PASC patients and non-PASC controls, respectively. Genes with a Benjamini–Hochberg adjusted *p* < 0.01 and an absolute value of log2(fold change) > 0.2 were deemed to be DEGs. Up-regulated DEGs are shown in red, while down-regulated DEGs are shown in blue. (**D**), Comparison of the composition of cell clusters across four groups. (**E**), Difference in the proportions of cell clusters across four groups).

T cells from those non-elderly female participants were further integrated with the CCA method. Through clustering and comparing expression patterns of cell marker genes, seven distinct CD4^+^ T cell clusters, including Treg, and five distinct CD8^+^ T cell clusters were identified (Figure 6A,B). We compared single-cell differential expression in CD4^+^ T and CD8^+^ T cells between PASC patients and non-PASC controls (Figure 6C). Many genes were significantly differentially expressed in CD4^+^ T and CD8^+^ T cells between PASC patients and non-PASC controls, including fewer ISGs, such as ISG20 and EPSTI1 (Figure 6C). There were obviously three increased CD4^+^ T cell subsets in PASC patients compared with non-PASC controls, namely, CD4c4, CD4c5, and CD4c6 (Figure 6D,E). The difference in the percentages of those T cell subsets between PASC and healthy controls was likely to be caused by the non-biological batch effect (Figure 6D,E). The outcomes of the comparison of T cell subset percentages between acute COVID-19 infection and PASC were shown in Supplementary Figure S2B, in which the differences may also be attributed to non-biological batch effects or technical variations across different study cohorts.

### 3.7. The scRNA-Seq Transcriptome Analysis of NK Cells

**Figure 7:**
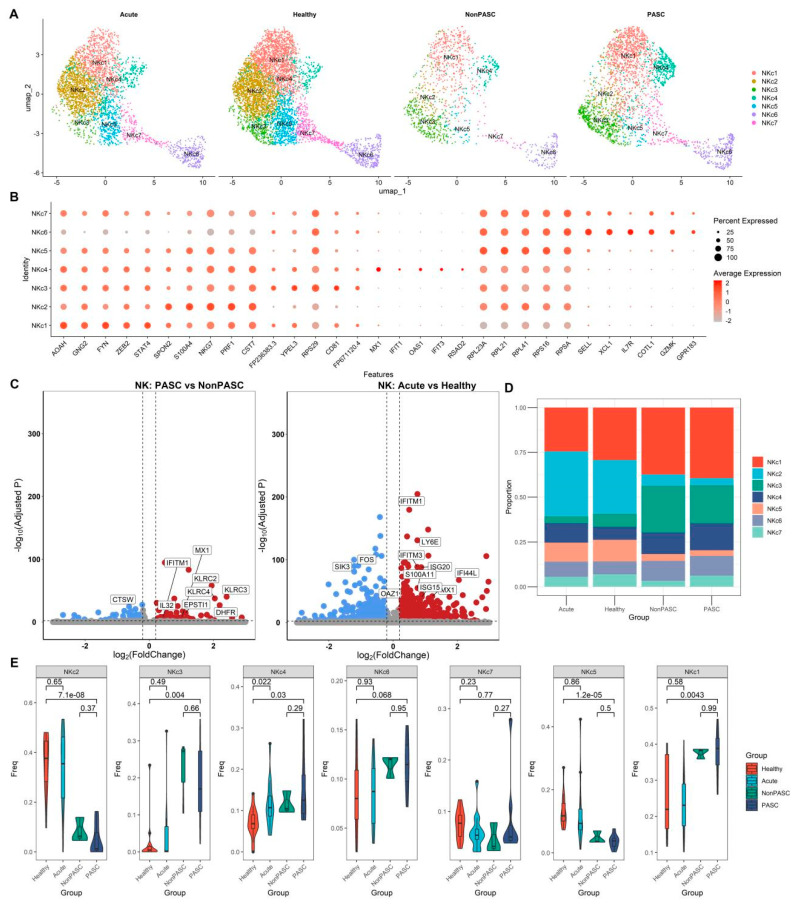
Single-cell transcriptome analyses of NK cells from non-elderly female participants consisting of acute COVID-19 infection patients, healthy controls, PASC patients, and non-PASC controls ((**A**), Cluster distribution via UMAP of NK cells across four groups. (**B**), Dot plot shows the expression percentages and the expression levels of key markers in different cell clusters. (**C**), Volcano plots for the DEGs in the NK cells between acute COVID-19 infection patients and healthy controls, and the DEGs in the NK cells between PASC patients and non-PASC controls, respectively. Genes with a Benjamini–Hochberg adjusted *p* < 0.01 and an absolute value of log2(fold change) > 0.2 were deemed to be DEGs. Up-regulated DEGs are shown in red, while down-regulated DEGs are shown in blue. (**D**), Comparison of the composition of cell clusters across four groups. (**E**), Difference in the proportions of cell clusters across four groups).

NK cells from those non-elderly female participants were further integrated with the CCA method. Seven distinct cell clusters were identified through clustering and gene expression patterns of cell markers (Figure 7A,B). Many genes were significantly differentially expressed in NK cells between PASC patients and non-PASC controls, but only a few ISGs were differentially expressed in NK cells, such as EPSTI1 (Figure 7C). There was no significant difference in the percentages of those NK cell subsets between PASC and non-PASC controls (Figure 7D,E). The difference in the percentages of those NK cell subsets between PASC and healthy controls was likely to be caused by the non-biological batch effect (Figure 7D,E). The outcomes of the comparison of NK cell subset percentages between acute COVID-19 infection and PASC are shown in Supplementary Figure S2C, in which the differences may also be attributed to non-biological batch effects or technical variations across different study cohorts.

## 4. Discussion

This study has identified the dysregulated immune cell clusters in non-elderly female patients with PASC by pooling scRNA-seq data, including monocyte, T cell, B cell, and NK cell. In particular, two monocyte subsets, including the CD14^+^STAT2^high^ cluster and the CD16c3 cluster, are found to be increased in PASC patients compared with non-PASC controls. The CD14 subset, characterized by high expression of AhR, is decreased in PASC patients compared with non-PASC controls. Currently available findings from several previously published studies have also supported monocytes as a key player in the development of PASC. A study has shown that antigen presentation by CD14^+^ monocytes is related to the persistent interferon-γ (IFN-γ) release of CD8^+^ T cells among PASC patients [15]. Accordingly, these findings have suggested the key roles of disease-specific monocyte clusters in the pathophysiological mechanisms of PASC, particularly the CD14^+^STAT2^high^ monocyte.

STAT2 is a transcription factor involved in the regulation of type I and III IFN signaling pathways [16]. It is not only an effector but a negative regulator of type I IFN signaling, playing a vital role in regulating the expression of ISGs [17,18]. Loss-of-inhibitory function mutation of STAT2 p. (A219V) leads to a sustained phosphorylation of STAT2 and the enhanced expression of ISGs, which thus causes type I interferonopathy due to a defective negative feedback regulation of type I IFN signaling [19]. It has been demonstrated that stat2 null mice exhibited defects in the type I IFN immune response and an elevated susceptibility to viral infection [20]. Human STAT2 deficiency can lead to the absence of STAT2-dependent types I and III IFN immunity, resulting in defects in antiviral immunity and a predisposition to severe viral infections [21]. Enhanced transcriptional activity of STAT2 can result in uncontrolled IFN-I signaling and type I interferonopathy [19,22]. The study by Boudewijns et al. has implicated that STAT2 signaling in hamsters played a key role in antiviral defense during SARS-CoV-2 infection, but it could also drive severe lung injury by promoting inflammation [23]. Therefore, STAT2 signaling plays an essential role during SARS-CoV-2 infection by regulating type I and III IFN immunity and the inflammatory response. In the present study, the TFs activity analyses have shown similar findings. We have identified STAT2 and IRF9 as the key TFs determining the functions of the two screened monocyte subsets (CD14^+^STAT2^high^ cluster and CD16c3 cluster), which are significantly increased in the non-elderly female patients with PASC. Furthermore, both the transcription activity and expression of STAT2 clusters are obviously enhanced in most peripheral monocytes from PASC patients compared with non-PASC controls. Some ISGs are found to be significantly upregulated in the featured monocytes from PASC patients, such as IFI6, IFITM3, IFI44L, IFI44, EPSTI1, ISG15, and MX1. Accordingly, STAT2-dependent signaling activation may play a critical role in the pathogenesis of PASC by regulating the types I and III IFN responses and the expression of common ISGs in monocytes. The precise effect and mechanism of the CD14^+^STAT2^high^ monocyte subset in PASC warrant investigation in the future.

PASC is a chronic disease following an acute SARS-CoV-2 infection with unclear pathophysiological mechanisms. It has been suggested that the pathophysiological mechanisms of PASC are multi-factorial, and different mechanistic subtypes of this disease may exist. The two most proposed pathophysiological mechanisms are the dysregulated production of inflammatory cytokines and the persistence of virus infection [24]. A previous study has suggested that PASC was associated with high expression of STING, cGAS, and IFN-α [25], which supports the findings in our study. In our study, increased expression of common ISGs has been identified in most peripheral monocyte subsets and other major immune cells from non-elderly female PASC patients, suggesting the crucial role of uncontrolled type I IFN signaling and/or type I interferonopathy in PASC. This finding provides new insights into the pathophysiological mechanisms of PASC.

Uncontrolled IFN-I signaling can result in severe interferonopathy, thus inducing a spectrum of diseases, such as systemic lupus erythematosus (SLE) [26], HIV infection [27], and COVID-19 [28]. Sex differences usually exist in COVID-19, which is characterized by uncontrolled IFN-I signaling. Several potential mechanisms have been proposed to explain the sex-specific pathophysiological processes in type I interferonopathy, including SARS-CoV-2 infection [29]. One key mechanism is the dosage of X-linked genes, such as TLR7 and TLR8 [27,30,31,32,33].TLR7 is encoded by the X chromosome and can escape X chromosome inactivation (XCI) with variable degree among different individuals, resulting in a bi-allelic expression of TLR7 in immune cells. Higher TLR7 expression can result in enhanced secretion of type I IFNs and increased expression of ISGs, thus promoting the development of type I interferonopathy. X-linked recessive TLR7 deficiency leads to about 1.8% of men under 60 years old suffering from life-threatening COVID-19 due to impaired type I IFNs in response to SARS-CoV-2 [34]. Furthermore, higher rates of severe status and mortality of COVID-19 have been found among male patients, suggesting a sex discrepancy in the outcome of COVID-19 [35]. The underlying mechanism is the sex discrepancy in the SARS-CoV-2 IgG antibody level, therefore resulting in different outcomes of COVID-19 between male and female patients. Furthermore, it has been reported that female COVID-19 patients are at an elevated risk of PASC compared with male patients [4,5,6,7]. PASC women need much longer to resolve. Also, the association of X-linked recessive TLR7 with type I interferonopathy was not observed in this study. Most importantly, the potential regulatory mechanisms of IFN-I signaling activation in PASC among non-elderly female patients warrant further elucidation in more future studies.

This study has shown PASC-specific monocyte subsets in the peripheral circulation. The expression of STAT2 was obviously enhanced in most peripheral blood monocyte subsets of PASC patients. In particular, this work highlights a distinctive CD14^+^STAT2^high^ monocyte subset in non-elderly female patients with PASC, emphasizing an overactive type I interferon pathway and a possible type I interferonopathy underlying the pathogenesis of PASC occurring among non-elderly female patients. From a clinical perspective, this uncontrolled interferon activation may contribute to persistent inflammation and immune injuries to tissues or organs, which are common in PASC [36]. The overactive type I interferon pathway observed in these patients can also intersect with complement system dysregulation and activation, which can further promote damage to normal tissues and thrombotic microangiopathy in PASC patients [37,38,39]. Thus, targeting the type I interferon pathway alongside complement modulation might offer novel therapeutic approaches for PASC.

There are limitations to the present study. This study has a risk of bias caused by non-biological batch effects or technical variations across different study cohorts. Most PASC patients and non-PASC controls were from the study cohort of GSE235050, while acute COVID-19 infection cases and healthy controls were from other study cohorts. Significant differences in the percentages of monocytes, B cells, and NK cells between PASC patients and healthy controls and significant differences in the percentages of NK cells between non-PASC controls and healthy controls were identified (Figure 1 and Figure S1), but those differences were likely to be caused by bias from non-biological batch effects or technical variations across different study cohorts. Furthermore, there were also significant differences in the percentages of B cells, monocytes, and NK cells between PASC patients and acute COVID-19 infection cases, which may also be attributed to non-biological batch effects (Supplementary Figures S1 and S2). The impact of these non-biological batch effects and technical variations could also significantly interfere with the outcomes of gene differential expression analyses, leading to a situation where only a subset of DEGs with initially substantial group differences retained statistical significance, while other DEGs did not show significant differences between groups in the presence of non-biological batch effects. The situation above made it challenging to determine the presence of true differential expression between the groups based on these disrupted results. Therefore, we mainly used outcomes of the comparison between the PASC group and the non-PASC group, which were from the same study cohort and could exclude the impact of those non-biological batch effects, and outcomes of the comparison between the acute infection group and the healthy control group, which had a low risk of non-biological batch effects. The outcomes of comparing the PASC group with acute COVID-19 infection cases and healthy controls need to be explored in future studies, in which non-biological batch effects and technical variations should be adequately controlled.

## 5. Conclusions

In summary, this study reveals several featured immune cells in the non-elderly female PASC patients, CD14^+^STAT2^high^ monocytes in particular. Uncontrolled IFN-I signaling activation and type I interferonopathy are revealed in PASC, which provides an updated understanding of the pathogenesis. More studies are warranted to clarify the roles and regulatory mechanisms of those PASC-specific immune cells in different pathophysiological processes and type I interferonopathy.

## Figures and Tables

**Table 1:** Characteristics of 41 females included in this study.

GSMID	Gender	Age (Year)	Group	Hospitalization	Sample Type	Reference
GSM7493832	Female	37	Non-PASC	No	PBMC	PMID: 38212464 [10]
GSM7493834	Female	38	Non-PASC	No	PBMC	PMID: 38212464 [10]
GSM7493835	Female	50	Non-PASC	No	PBMC	PMID: 38212464 [10]
GSM7493836	Female	57	PASC	No	PBMC	PMID: 38212464 [10]
GSM7493837	Female	46	PASC	Yes	PBMC	PMID: 38212464 [10]
GSM7493838	Female	49	PASC	Yes	PBMC	PMID: 38212464 [10]
GSM7493839	Female	33	PASC	No	PBMC	PMID: 38212464 [10]
GSM7493840	Female	43	PASC	Yes	PBMC	PMID: 38212464 [10]
GSM7493841	Female	48	PASC	No	PBMC	PMID: 38212464 [10]
GSM7493842	Female	26	PASC	No	PBMC	PMID: 38212464 [10]
GSM7493843	Female	46	PASC	Yes	PBMC	PMID: 38212464 [10]
GSM6249254	Female	29	Acute COVID-19	Not provided	PBMC	PMID: 35810186 [8]
GSM6249257	Female	32	Acute COVID-19	Not provided	PBMC	PMID: 35810186 [8]
GSM6249270	Female	45	Acute COVID-19	Not provided	PBMC	PMID: 35810186 [8]
GSM6249272	Female	27	Healthy	--	PBMC	PMID: 35810186 [8]
GSM6249274	Female	44	Healthy	--	PBMC	PMID: 35810186 [8]
GSM6249276	Female	60	Healthy	--	PBMC	PMID: 35810186 [8]
GSM4509015	Female	63	Healthy	--	PBMC	PMID: 32651212 [9]
GSM4509022	Female	38	Acute COVID-19	Not provided	PBMC	PMID: 32651212 [9]
GSM4509023	Female	54	Healthy	--	PBMC	PMID: 32651212 [9]
GSM4697613	Female	53	Acute COVID-19	Yes	PBMC	PMID: 35064122 [12]
GSM4697623	Female	56	Acute COVID-19	Yes	PBMC	PMID: 35064122 [12]
GSM5025734	Female	46	Acute COVID-19	Not provided	PBMC	PMID: 35281000 [11]
GSM5025736	Female	53	Acute COVID-19	Not provided	PBMC	PMID: 35281000 [11]
GSM5025737	Female	39	Acute COVID-19	Not provided	PBMC	PMID: 35281000 [11]
GSM5025739	Female	47	Acute COVID-19	Not provided	PBMC	PMID: 35281000 [11]
GSM5025741	Female	53	Acute COVID-19	Not provided	PBMC	PMID: 35281000 [11]
GSM5025742	Female	61	Acute COVID-19	Not provided	PBMC	PMID: 35281000 [11]
GSM5025744	Female	46	Acute COVID-19	Not provided	PBMC	PMID: 35281000 [11]
GSM5025764	Female	53	Acute COVID-19	Yes	PBMC	PMID: 35281000 [11]
GSM5025769	Female	38	Acute COVID-19	Yes	PBMC	PMID: 35281000 [11]
GSM5025770	Female	44	Healthy	--	PBMC	PMID: 35281000 [11]
GSM5025771	Female	34	Healthy	--	PBMC	PMID: 35281000 [11]
GSM5025772	Female	39	Healthy	--	PBMC	PMID: 35281000 [11]
GSM5025777	Female	50	Healthy	--	PBMC	PMID: 35281000 [11]
GSM5025778	Female	52	Healthy	--	PBMC	PMID: 35281000 [11]
GSM5025780	Female	56	Healthy	--	PBMC	PMID: 35281000 [11]
GSM7512193	Female	61	Healthy	--	PBMC	PMID: 38259488 [13]
GSM8123915	Female	25–50	Healthy	--	PBMC	PMID: 38509155 [14]
GSM8123910	Female	25–50	Healthy	--	PBMC	PMID: 38509155 [14]
GSM8123895	Female	25–50	Healthy	--	PBMC	PMID: 38509155 [14]

## Data Availability

All datasets generated for this study are included in the article.

## Supplementary Materials

The following supporting information can be downloaded at: https://www.mdpi.com/article/10.3390/v16091369/s1, Figure S1: Difference in the proportions of major immune cells across four groups or the proportions of monocyte subset clusters across four groups ((A), Difference in the proportions of major immune cells across four groups. (B), Difference in the proportions of monocyte subset clusters across four groups). Figure S2: Difference in the proportions of cell subset clusters across four groups ((A), Difference in the proportions of B cell subset clusters across four groups. (B), Difference in the proportions of T cell subset clusters across four groups. (C), Difference in the proportions of NK cell subset clusters across four groups).
