# Supplementary material for: Type I Interferonopathy among Non-Elderly Female Patients with Post-Acute Sequelae of COVID-19
Source: Viruses. 2024 Aug 28;16(9):1369. doi: 10.3390/v16091369 (PMC11435747; doi:10.3390/v16091369)
Supplement: Supplementary file 1 [file viruses-16-01369-s001.zip › viruses-3155892-supplementary.pdf]

Type I Interferonopathy among Non-elderly Female Patients with Post-Acute Sequelae of COVID-19

Donghua Xu[1, 2], Xuebin Qin[1, 2, *]

Legends of Supplementary figures

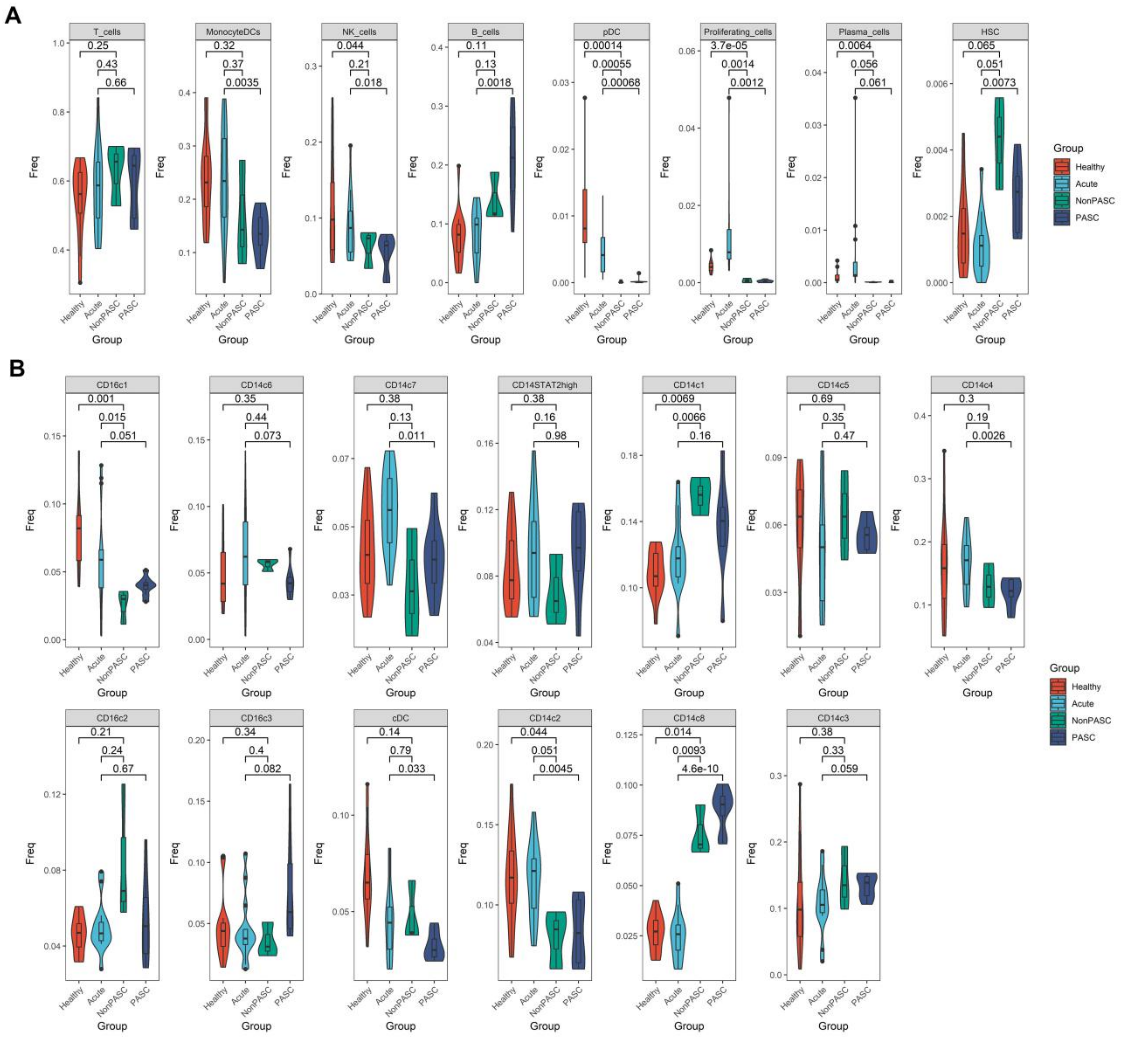

Supplementary figure S1 Difference in the proportions of major immune cells across 4 groups or the proportions of monocyte subset clusters across 4 groups (A, Difference in the proportions of major immune cells across 4 groups. B, Difference in the proportions of monocyte subset clusters across 4 groups.)

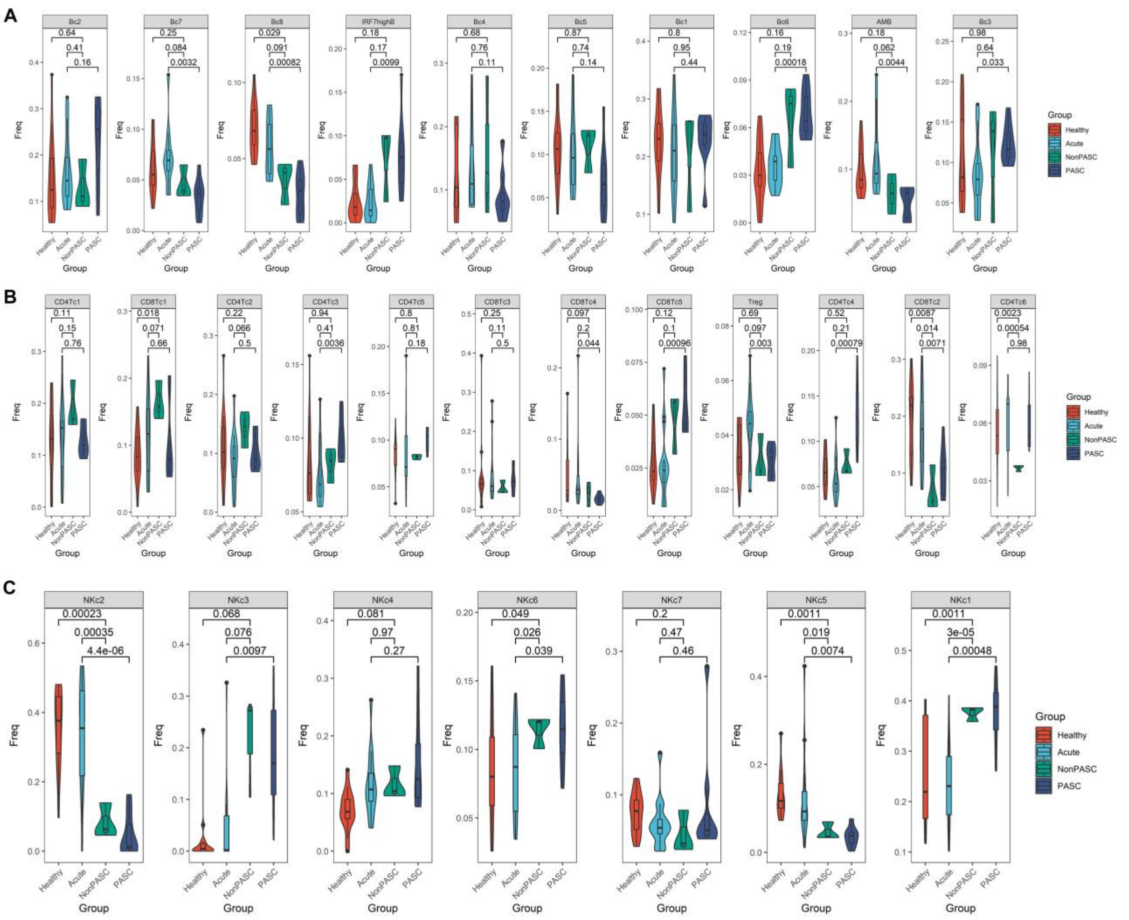

Supplementary figure S2 Difference in the proportions of cell subset clusters across 4 groups (A, Difference in the proportions of B cell subset clusters across 4 groups. B, Difference in the proportions of T cell subset clusters across 4 groups. C, Difference in the proportions of NK cell subset clusters across 4 groups.)
